# The Child as Vulnerable Victim: Humanitarianism Constructs Its Object

**DOI:** 10.3390/ijerph20065102

**Published:** 2023-03-14

**Authors:** Jason Hart

**Affiliations:** Department of Social and Policy Sciences, University of Bath, Bath BA2 7AY, UK; jh462@bath.ac.uk

**Keywords:** vulnerability, children, humanitarianism, trauma, agency, participatory programming, psychosocial programming

## Abstract

Over the last one hundred years, humanitarian agencies have considered children primarily through the lens of vulnerability. Advocacy for attention to children’s agency and for their participation has burgeoned since the 1980s without shifting the powerful hold that assumptions of vulnerability have had over the policy and practices of humanitarians. This article seeks to denaturalise the conceptualisation of children in contexts of emergency as primarily vulnerable (would-be) victims, placing it in historical and geopolitical contexts. It offers a critical analysis of both conventional humanitarian thinking about vulnerability per se and the reasons for its continued invocation in settings of displacement and political violence. Drawing upon examples from the Mau Mau rebellion against British colonial rule in 1950s Kenya, and current humanitarian response to the situation of Palestinian children living under Israeli occupation, this article relates the continued dominance of the vulnerability paradigm to the pursuit of self-interest by elites and the survival strategies of humanitarian agencies. It pays particular attention to the uses to which mental health thinking and programming is put in what may be called the ‘politics of pathologisation’.

## 1. Introduction

**Figure 1:**
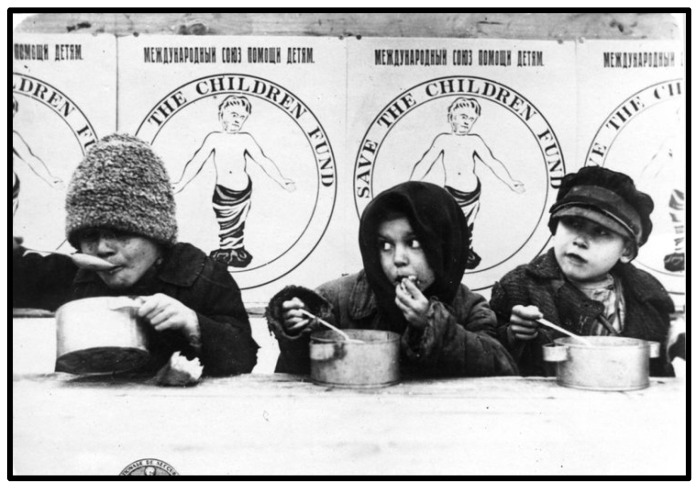
Famine in Russia, 1921–1923. Save the Children. All rights reserved.

For more than a century, children have been an explicit focus of humanitarian institutions. In 1919, one of the very first major non-governmental aid agencies—Save the Children Fund (SCF)—was founded specifically to attend to the needs of the young caught up in warfare. This event followed swiftly on the heels of a campaign to lift the blockade of Germany and Austria so that food and medical supplies could reach sick and starving children. The campaign sought to strengthen concern for the young in order to counter popular opposition to feeding ‘children of the enemy’ ([1] p. 273). The image of the child as a vulnerable and universal victim was central to SCF’s efforts to garner support for humanitarian efforts transcending national borders and nationalistic sentiments. In its early years, SCF’s logo emphasised vulnerability through the image of an infant in swaddling with arms outstretched and eyes downcast (Figure 1 below).

In developing a discourse of vulnerability, SCF drew upon a conceptualisation of the young that had grown in prominence since the 18th Century. Societal change in Western Europe and North America, influenced by the Romantic movement, had steadily oriented popular understanding towards a vision of the child as an innocent being, requiring separation from the adult world to avoid contamination. Vulnerability—physical, psychological, and moral—was the quality most particularly invoked to justify the intervention of caring adults. Without intervention, victimhood and unconscionable suffering were likely, if not inevitable.

The conceptualisation of children as, first and foremost, vulnerable potential victims served the efforts of SCF well at the moment of its inception in the wake of the first World War. Once firmly established, this understanding remained fundamentally unchanged until today. More than a hundred years after the establishment of SCF, children in humanitarian settings are still represented and discussed as, above all, vulnerable victims.

Such endurance of the conceptualisation of children merits investigation in terms of its drivers. It is especially curious in light of calls by humanitarian professionals and scholars over recent decades to acknowledge children’s capacity to act meaningfully upon their circumstances. Theorised and conveyed in the language of agency and pursued on the ground through ‘participatory’ programming, humanitarians have increasingly sought to engage children as social actors. Yet vulnerability remains the dominant view in humanitarian settings, invoked to justify limiting efforts to engage the young as social actors within their families and communities.

**Figure 2:**
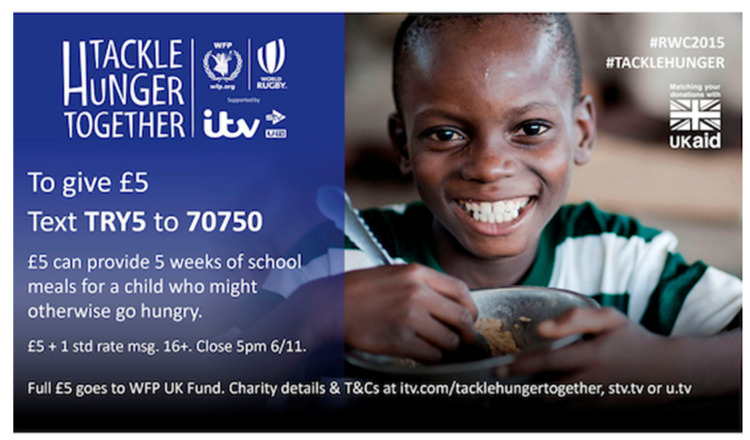
UK Funding Appeal for 2015 Campaign.

The attribution of vulnerability as the primary quality of children is not limited to humanitarian settings. Indeed, the UN Convention on the Rights of the Child—to which all but one UN member state is a full signatory—has served to promote this view of the young across the globe. However, the reasons for perceiving the young through the lens of vulnerability has particular drivers and particular consequences in settings of humanitarian emergency. The enduring—even growing—dominance of this view may be attributed to numerous factors. They include popular perception and reaction. In the 1990s, humanitarian agencies responded to criticism that their depiction of children within fund-raising appeals as distressed victims was degrading. More positive and agentive imagery was employed [2]. Thus, for example, in the place of images of evidently starving children, agencies depicted smiling beneficiaries of food aid (Figure 2 below).

In recent years, however, there has been a return to previous practice in light of evidence that depiction of victimhood and damage is more likely to elicit support from the general public for aid initiatives. (I prefer not to perpetuate the use of such imagery. However, a simple internet search will lead readers to multiple images of funding campaigns that relate to humanitarian efforts in many parts of the world. At the current time, funding appeals for Yemen seem particularly to rely on shocking images of children in extreme distress.) Moreover, there is implicit understanding amongst humanitarians that it is often in children’s own interests to be addressed and represented as vulnerable victims. This may be the case, for example, following demobilisation from military groups when children’s vulnerability is invoked to mitigate the punishment meted out to former soldiers (see, for example, [3]). However, the opposite might also be true. When the young are imagined as passive, vulnerable victims, their insights, and their actions to protect themselves are unlikely to be recognised. Ignored on the matters that affect them and their strategies for survival, the threats to children in humanitarian settings may increase. For example, treating girls who wed young in situations of conflict or displacement simply as vulnerable victims may blind us to their employment of marriage as a protection strategy, and thereby undermine them [4]. Similarly, assuming that a child of, say, 13 or 14 years is incapable of taking care of a younger sibling in a setting where adult care is unavailable can close off the opportunity to be heard on the support that child needs to fulfill their responsibility. Thus, it is questionable whether invoking vulnerability and victimhood while obscuring the need to engage with children as agentive amidst the worst of situations necessarily serves their best interests.

Aside from the utility of the vulnerable victim trope, there are further reasons for its continued employment. In this paper, I attend particularly to one such reason: the pursuit of self-interest by political/economic elites. I explore how depiction of the young primarily as vulnerable would-be victims serves the agendas of governments, corporations, and even humanitarian agencies. To illustrate the discussion, I draw upon the work of Emily Baughan concerning SCF’s role in the Mau Mau rebellion of the early 1950s and on my own exploration of humanitarian efforts in the Palestinian territory occupied by Israel since 1967. In their different ways, both situations illustrate the link between the treatment of children as vulnerable and traumatised, on one hand, and elite interests, on the other. Furthermore, both situations reveal the strategising of child-focused humanitarian organisations, negotiating a path between their mandate for children’s protection, on one hand, and their own institutional advantage, on the other.

## 2. The Pre-History of the Vulnerable Humanitarian Child

The peculiarity of the stasis in conceptualisation of children as primarily vulnerable beings within humanitarian effort is starkly revealed when we consider the ongoing evolution in ideas about children witnessed in Europe and North America between the mid-18th and early 20th centuries. I offer a brief overview of this process of change to demonstrate both the geographical specificity of such a conceptualisation as well as its historical contingency. In this way, I seek to de-essentialise the current hegemonic view of children as vulnerable victims within the field of humanitarianism. This is necessary for the development of protection work more relevant to the context and lives of particular populations of children living amidst humanitarian emergency.

According to Hugh Cunningham, during the 1700s a profound shift in ideas about children occurred:

“Children can be classed alongside slaves and animals as the recipients of the sentimentalism and humanitarianism that characterized the latter part of the eighteenth century…The art of child-rearing became one of harkening to nature, giving free reign to growth, rather than bending twigs to a desired shape.”

([5] pp. 61–62)

This shift in perception should be understood within wider processes of change taking place in Western nations at that time. This was the age of Enlightenment thought in which the dominant role of Christian institutions and religious doctrine in people’s lives and thinking was subject to unprecedented challenge. With the consequent, if uneven, decline of the authority of the Church children were ‘transformed from being corrupt and innately evil to being angels, messengers from God to a tired adult world.’ (Ibid, p. 62) While the register may still have been religious, the source of such a shift in thinking about children was secular—most particularly Romantic poets, novelists, and philosophers such as Rousseau, Wordsworth, Goethe, and Hugo.

Such change in popular imagination did not happen uniformly across societies, however. Nor was it achieved without contestation. For example, 19th Century Evangelicals gave new life to the Puritan view, familiar in the preceding two centuries, of the inherent sinfulness of the young. This belief directly countered the image of childhood innocence and, while it did not ultimately prevail, its articulation through this period illustrates the contingency in conceptualisation of the young [6].

Together with innocence, the quality that came most particularly to be attributed to children was vulnerability. To the citizenry of late 18th and early 19th Century France, England, Sweden, and other European countries, the evidence of children’s physical vulnerability was only too apparent. Serious illness and accidents were plentiful, especially in the rapidly growing cities, and in the factories and cotton mills where working-class children toiled ([5] p. 90).

Despite the fall in child mortality rates in the late 19th Century, the view of children as especially vulnerable endured. Moreover, they were increasingly seen as dependent upon adults and in need of their protection: a view reinforced by children’s removal from factory labour that brought to an end the standing that many enjoyed as contributive economic actors [7]. Citizens in Britain, the United States and other Western nations established a range of organisations concerned explicitly with child protection. The most notable of these was the National Society for the Prevention of Cruelty to Children (NSPCC), the first branch of which was created in New York in 1875.

The forces—philosophical, artistic, economic, and social—that nurtured and promoted a view of children as vulnerable, emerged in the 18th Century and endured through the 19th. During this period, ideas and information had to a limited extent been exchanged across national boundaries, contributing to broadly simultaneous processes of change within numerous European and North American nations. However, at the turn of the 20th Century dialogue between those working for children’s protection and welfare took on a fully transnational character. This was a vital step towards the instantiation of the vulnerable child victim as an international object of institutionalised humanitarian effort.

## 3. World War One and The Emergence of Child-Focused Humanitarianism

In the view of Cabanes, World War One was “an unprecedented catastrophe” for the people of Europe ([1] p. 1). According to him, the experience of this war.

“…fostered a deep and long-term pacifist feeling among a substantial population, and it made the protection of all the war’s victims, civilians, and soldiers alike, an absolute necessity–a project that drew to it a surprisingly large and talented group of activists and their supporters.”

([1] p. 1)

Some of these activists had witnessed the war at close quarters. Most came from the upper echelons of their respective societies yet generally operated as private individuals: apart from or even antagonistic towards their governments. Together with dedicated fellow campaigners, they strove to establish a global ethos for humanitarianism as well as new national and international institutions that would work to provide relief on the ground. In the estimation of Cabanes, the immediate post-war period constituted “a humanitarian moment–that is, a period of several years in which important collective expectations were consolidated.” ([1] p. 16)

The production of a particular ontology of the child as the object of protection efforts may be seen as one of the ‘collective expectations’ that were ‘consolidated’ during the immediate post-World War One period. The characteristic of vulnerability attributed to this object had been naturalised through changes that occurred over a period of roughly two centuries. Now they served to motivate and shape humanitarian action. Appeals launched to engage the public and parliamentarians in support of measures intended to ameliorate the conditions of children in post-WW1 Europe traded in imagery of children in distress ([8] p. 133). The images were predominantly of younger children, and they were employed in both visual and textual material. In a direct challenge to the nationalistic concerns expressed about the children of enemies, SCF sought to promote a universal notion of the child as innocent victim, compassion for whom was ‘natural’ and transcended national borders. To quote Baughan and Fiori:

“…the organisation’s actions were presented as…an expression of innate human sympathy for vulnerable children.”

([8] p. 131)

In the wake of the First World War, with its destruction of civilian life and infrastructure, SCF was the first globally oriented, non-governmental humanitarian agency to be established. Numerous other organisations came into being in response to subsequent emergencies, particularly in the period around World War Two. For example, Plan International was founded in response to the Spanish Civil War (1936–1939); the Greek Famine of the early 1940s prompted the creation of Oxfam; and the Korean War (1950–1953) provided the stimulus for the establishment of WorldVision. These and numerous other international NGOs constitute a field of humanitarian activity that may be distinguished by particular characteristics. Following Redfield and Bornstein, I take these defining characteristics to be (1) western-originating, (2) institutionalised and professionalised, and (3) secular in orientation [9]. It is this form of humanitarian endeavour that has been witnessed globally over the last century and more, animated principally by a bourgeois, European/North American world view, including a particular view of children. There is nothing inevitable about either this form of humanitarianism or the vision of the child that it promotes and reproduces. Both are artefacts of societal forces that have evolved over time and across context, not least of which is the pressure upon humanitarian agencies to ensure their own institutional survival within a crowded marketplace in which political-economic elites, in government and beyond, hold the purse strings.

## 4. The Conceptualisation of Vulnerability

Vulnerability is a term understood differently across a range of debates and processes within the humanitarian field. The work of child-focused humanitarians to achieve the protection and wellbeing of the young is predicated upon an understanding of vulnerability that has two distinguishing features. The first of these is the contention that vulnerability is a universal and undifferentiated property of children as children. Humanitarian organisations have promoted a universalistic view of the child as a being under the age of 18, in line with the definition specified in the UN Convention on the Rights of the Child. Beyond the eighteenth birthday lies adulthood.

Dividing humanity rigidly between ‘child’ and ‘adult’ has been accompanied by the frequent homogenisation of ‘childhood’ experience that insufficiently attends to change across the first eighteen years of human life. Vulnerability is attributed to all under 18-year-olds, ignoring, for example, differences between young children and teenagers. Moreover, age in itself is not the only factor that may produce significant difference. The importance of analysis of children’s situation that also embraces class, race/ethnicity, sexual orientation, (dis)ability, and gender—individually and in intersection—is becoming a discussion point within the academic literature [10,11]. However, such concern is yet to find much traction within humanitarian practice. We may take as an example a 2022 UNHCR report on refugees in Jordan which employed that organisation’s own Vulnerability Assessment Framework introduced in 2014 [11]. Child labour is one of the issues around which data were collected for the report. Gendered difference is briefly noted (more boys involved in labour than girls) and data are presented on working children with disabilities. However, beyond this no meaningful consideration is given to intersectionality. Indeed, even age is given little consideration: 5–15-year-olds are treated as a single group. While a division between ‘Syrian’ and ‘non-Syrian’ refugees is utilised, no attention is given to the impact of nationality either, let alone race/ethnicity—a significant omission for reasons explained below.

The second distinguishing feature of humanitarians’ understanding of vulnerability is its treatment as an inherent property. Typically in assessing vulnerabilty children are conceptualized as a single category from 0–17 years of age and labelled with such terms as “non-autonomous family members” ([12] p. 39). An alternative view is that societal factors have significant impact upon the ways and extent that children are rendered vulnerable—a point reinforced by ethnographic studies of the lives of children in contexts of political violence and displacement [13,14]. The view of vulnerability as an inherent property is easier to pursue in practice since it justifies an approach that attributes vulnerability a priori on the basis of categories (the young, the elderly, disabled, LGBTQ+, etc.). On-the-ground analysis to identify who is vulnerable and why is thereby rendered unnecessary by such a ‘group’ approach. Furthermore, by locating the cause of vulnerability in the person or in their immediate situation—such as being a member of a ‘Female Headed Household’ [15]—wider social, economic, and political forces that render people vulnerable are kept out of view. Numerous societal phenomena, such as racism, inequality, patriarchy, structural violence, may each serve to render children vulnerable to harm, but they are unlikely to be considered, let alone confronted, when vulnerability is seen as an inevitable consequence of being young.

To illustrate both the importance of an intersectional approach and of seeing vulnerability as, at least in part, a product of context, I cite a study conducted in 2021–2022 involving a team of academics, including myself, and members of refugee communities that explored the protection risks faced by refugee children from four countries (Somalia, Sudan, Iraq, and Syria) now living in Jordan. (The team included Mohammed Alruzzi, Caitlin Procter and Kirsten Pontalti as well as ‘peer researchers’ from the Sudanese, Somali, Syrian and Iraqi refugee communities in Amman recruited by the organisations Sawiyan and Collateral Repair Project.) Such risks were not uniform in either kind or scale across these different nationalities. Indeed, nationality itself was a factor mediating children’s vulnerability given the variation in support and assistance that host government and humanitarian actors provided based on country of origin. Skin colour was also a major issue given the pervasive racism/‘colourism’ within Jordanian society. Children from Africa were consequently vulnerable to bullying and interpersonal violence to an extent not shared by their Syrian and Iraqi peers. The harm to which Somali and Sudanese were routinely exposed took forms that differed depending on gender, age, disability (including learning difficulties) and proficiency in the Arabic language. (Some Somali refugees came to Jordan after many years living in other Arab countries in the region and thus spoke Arabic. However, those who came directly from Somalia and did not speak the language were vulnerable to additional forms and degrees of harm that distinguished them from the other three national groups). Employing an intersectional lens, the research revealed how, for example, a 15-year-old Somali boy was vulnerable to multiple forms of harm that differed from the harms experienced by a 10-year-old Syrian girl. To give a specific example of the former, several caregivers in the Somali community spoke of the bullying and violence experienced by their teenage boys at school. As they explained, a combination of lack of formal status within Jordan, systemic racism, and anti-migrant sentiment discouraged them from seeking assistance from the police even in cases of violent, interpersonal attack. Thus, it was not the fact of being young, or black, or lacking the status of ‘refugee’ that in themselves created vulnerability. Rather it was the societal response to these aspects of individual identity.

The choice to depict children in humanitarian settings as inherently vulnerable rather than as rendered vulnerable by circumstances is profoundly political in its implications, if not its intention. The ascription of vulnerability as an inherent property draws attention away from the specific dynamics of the setting, such as government or humanitarian agency policy. By contrast, a view of vulnerability as the product of interaction between children and their environment urges consideration of the need to challenge discrimination, political and economic marginalisation, and the racist, gerontocratic and patriarchal exercise of power.

Depicting vulnerability as an inherent property of children is routine within the child-focused humanitarian field. Understood in this way, assessment of actual children is not required in order to label them as vulnerable. If you are young in a humanitarian setting, then vulnerability and (potential) victim are typically deemed inevitable. Working in Nepal in the early 2000s, I witnessed the hold of such thinking over child-focused humanitarians. During the 1990s, international and local NGOs had established a large number of children’s clubs in villages across Nepal that gained global recognition as a model of meaningful child participation and children’s agency. Throughout that period, government forces were engaged in armed conflict with Maoist rebels in what the latter called ‘the People’s War’. Mindful of the Nepali government’s sensitivities, UN agencies and most INGOs proceeded with development, rather than humanitarian, work acting as if the conflict was not happening.

Around the time of my work in Nepal—in 2001 and 2002—military activity flared up and the Nepali Government became less reticent about acknowledging the events unfolding. Outside agencies consequently switched into humanitarian mode. Within this reconceived terrain, one of the largest child-focused agencies began to train ‘para-psychologists’ to go into the field in order to assess the mental state of children and offer support. Those who were to receive this intervention were the same children who, hitherto, has been praised for their great contribution to transforming their villages. Now these admirable ‘social actors’ began to be treated as vulnerable victims. All that had changed was framing of the space by humanitarian organisations. This led to an unreflexive flip in the conceptualisation of ‘child’ from agentive social actor to vulnerable victim. Greater nuance was introduced further down the line, but the initial response betrayed the reductive and formulaic way that the young are commonly conceptualised by aid organisations: child + humanitarian crisis = vulnerability.

## 5. Agency and Vulnerability

To some extent, there is now a willingness to acknowledge children’s agency during humanitarian crisis: albeit at particular moments and in specified spaces. The perception of the young as social actors was increasingly embraced in the 1990s by agencies doing development work. From there, the notion gradually got taken up within the humanitarian field. Conviction in children’s capacity to act upon their circumstances, even in the midst of emergency, and to contribute to their families, communities, and societies, has typically translated into institutionally managed processes of participation in which the young are given the opportunity to articulate their experiences, needs and aspirations. The current enthusiasm for this approach is witnessed in the five-year strategy (2021–2025) of the Alliance for Child Protection in Humanitarian Action where the need for children’s ‘meaningful participation’ is highlighted repeatedly, even to the point of calling for a ‘child participation revolution’ ([16] p. 20).

The advocacy for children in humanitarian contexts to be treated as agentive social actors was propelled by the project of children’s rights as it grew in the latter decades of the 20th Century. In 1989, the United Nations introduced into international law the Convention on the Rights of the Child (UNCRC). This was the third iteration of a global charter on children’s rights beginning with the 1924 League of Nations Declaration on the Rights of the Child, followed by the 1959 UN Declaration. However, it was the first to advance a view of the young as social actors with the capacity—and right—to voice their concerns, particularly in matters that affect them, and for those concerns to be taken seriously.

Around the same time as the appearance of the Convention, scholars principally from the disciplines of sociology, anthropology and human geography offered a concerted challenge to the hitherto orthodox view—promoted by certain influential scholars in the fields of developmental psychology and pedagogy—of the young as passive objects of adult-led processes of socialisation, contributing little to their own development [17,18]. A foundational text within Childhood Studies—a multidisciplinary field that grew in prominence through the 1990s—argued instead that:

“Children are and must be seen as active in the construction and determination of their own social lives, the lives of those around them and of the societies in which they live. Children are not just the passive subjects of social structures and processes.”

([19] p. 8)

The proposition that children are agentive social actors seems to challenge the view of them as primarily vulnerable would-be victims. However, within the humanitarian field the acknowledgement of agency is intricately bound up with the continuation of victimhood as the dominant trope. In practice, the acknowledgement and encouragement of children’s exercise of agency is conditioned by the perception of potential victimhood: if humanitarians perceive risk, then children’s participation is suspended or, at least, managed in a manner that seeks to minimise harm. In the process, observers are reminded that children—all children—are, before any other consideration, inherently vulnerable.

Humanitarians have advanced a view of children as agentive and, at the same time, sought to set boundaries for the exercise of that agency. Children are acknowledged as social actors in situations that fit the norms of agencies and their staff. Where their actions run counter to those norms, agency is denied or ignored. Thus, for example, children who participate in activities focused on peace are typically lauded as social actors by humanitarians (For example, this from WorldVision Available online: https://www.wvi.org/peacebuilding-and-conflict-sensitivity/empowering-children-peacebuilders, accessed on 3 October 2023. Also [20]). Conversely, those who engage in political protest, especially when this involves some form of violence, or sexual activity, are routinely labelled as victims whose inherent naivety is being exploited by unscrupulous adults [21]. The principle of ‘best interests’ (Article 3), which is central to the UNCRC, can be invoked by humanitarians to justify curtailment.

How engagement with children is pursued has potentially serious implications for humanitarian organisations. Interactions that embrace children’s roles and views in an expansive manner can attract the charge that neutrality has been abandoned. To avoid offending the sensitivities of donors—governmental and corporate—careful management of interaction with the young may be deemed vital by humanitarians. This issue is explored below in discussion of humanitarian response to children in the occupied Palestinian territory.

## 6. From Societal Participation to Agency-Managed Participation

Within the UK, Europe, and North America, there are numerous historical examples of children’s engagement that include not just the economic and social domains but the political and military as well. Such participation by the young is underpinned by recognition of their capacity to contribute to family, community, and nation. For example, in Britain the many teenage boy soldiers in World War One were widely cheered as brave patriots [22,23]. Children’s involvement in the anti-apartheid struggle from the 1970s onwards was viewed at that time as legitimate political activism by aware young people [24]. The participation by boys and girls in violent acts of resistance in Nazi-occupied Europe during World War Two has been similarly lauded and memorialised [21].

By contrast, accounts of children’s involvement in military roles in the last quarter century have focused overwhelmingly on brainwashing and exploitation by unscrupulous leaders, ignoring the accounts of the young themselves that do not support such a view [25]. Children associated with groups engaged in political violence have been comprehensively depicted as vulnerable victims and their activities pathologised [26]. According to David Rosen, this demonstrates humanitarian organisations’ fundamental association of children with vulnerability and victimhood:

“…nearly all humanitarian and human rights efforts on the child soldier issue are shaped by a common belief that all persons under age eighteen are particularly vulnerable and innocent and that modern warfare is especially horrible. Though barely rooted in fact, these core beliefs profoundly shape the international conversation about child soldiers. Advocates for banning child recruitment equate childhood with vulnerability, and recognizing this equation is central to understanding humanitarian views of the child soldier.”

([21] p. 77)

While there has been a discernible change over time towards the increasing pathologisation of (older) children’s participation as activists and combatants, it is important not to see this simply as a steady and consistent evolution. Political considerations produced a view of the young in certain conflict-affected settings as vulnerable victims even while their peers elsewhere were being lauded for their heroism. A powerful example is provided by Emily Baughan in her account of the pathologisation of Kenyan boys involved in the Mau Mau rebellion of the 1950s against British colonialism. This rebellion was a response to the extreme predations of white settlers on farming land that had resulted in displacement, poverty, and exploitation:

“The vast farms owned by white settlers led to severe land shortages for Africans. Young Kikuyu were unable to attain the resources needed to begin new family units and become economically independent from their elders, and they felt this land shortage acutely.”

([27] p. 161)

As part of the response to violent uprising, Save the Children joined forces with the British colonial authorities. Framed as an effort to ‘rehabilitate’, adolescent rebels were held in centres that were “prisons in all but name” ([28] p. 163). In these facilities, inmates were taught skills and encouraged to develop dispositions that would serve them in building livelihoods not dependent on access to land.

Both SCF and the colonial government conjured a moral narrative of child rescue to justify their imprisonment of thousands of, mostly male, Kikuyu people. Underwriting this justification was an assumption that the young were victims whose inherent vulnerability had been exploited by the adult leaders of the Mau Mau rebellion. This was a narrative that could be used to garner support from the British public.

“Working with the internationally renowned SCF, the colonial government framed its reforms as progressive and compassionate. Humanitarian intervention lent legitimacy to the incarceration of teenage boys and the wider campaign against the Mau Mau.”

([28] p. 166)

Portraying the incarceration and maltreatment of thousands of boys as progressive and compassionate was made possible by implicit denial of their agency and by their depiction as “victims of Mau Mau” (Ibid). In this way, the colonial project in Kenya, of which British elites were the greatest beneficiaries, could also be framed as a moral project in which foreign institutions sought to save vulnerable boys from the savagery of their own society. This example illustrates the need to attend to the wider political context in which claims of children’s vulnerability are being made, and to enquire into the nature of the connection between humanitarian organisations and powerful political and economic actors.

The historical example of SCF’s involvement with the British colonial authorities in suppressing the Mau Mau rebellion is also an early example of the pathologisation of the young for political purposes. The contribution of SCF in this situation offered a foretaste of the mental health framework that has become so pronounced in humanitarian responses to children around the globe. It is to the connection between vulnerability and mental health that we now turn.

## 7. Mental Health and Vulnerability

Commonly referred to as ‘psychosocial programming’ and designed, if not led, by experts from Europe and North America, mental health as a domain of activity has grown in importance since the 1990s to become a core element of child-focused programming in many humanitarian settings [29]. In the early years, psychosocial activity focused on trauma and healing [30]. More recently, western-designed programmes have increasingly been framed as the means to promote resilience [31,32]. Both the trauma and resilience models tend towards simplification, however. Children are reduced conceptually to damaged victims or plucky survivors whose resilience is built via some form of psychosocial intervention. They are typically conceptualised and treated in a manner that divorces them from their communities. This process has been linked to neoliberalism with its individuating ethos [33].

An alternative approach might relate children’s mental wellbeing to the delivery of decent services, creating economic opportunity, tackling oppression, and achieving justice. However, a multi-faceted, grounded and more holistic approach is uncommon in psychosocial programming as designed by staff in the HQs of child-focused humanitarian agencies in the global North. By contrast, there are examples of mental health and wellbeing initiatives in the global South designed and led by locals that bring together a focus upon material conditions, justice, and healing [34].

As Pupavac has described, within the popular imagining of British society since World War Two refugees have been transformed from “self-determining political subjects” to “vulnerable survivors” who require intervention to address their trauma ([35] pp. 274 & 272). Furthermore, it is through evidence of trauma that the legitimacy of an individual’s claim for asylum and the associated protection and support is substantiated ([35] p. 278). This is particularly evident in the case of children. In her work on the treatment of unaccompanied children within the age assessment processes of the UK asylum system, Crawley describes the script to which the young should conform in order to meet the expectations of border officers regarding their age [36]. It is a script that entails the demonstration of damage and trauma. In such a situation, it is, paradoxically, the most aware and resourceful children who may do best in in presenting themselves in a manner that confirms to officials’ expectations of victimhood.

## 8. Trauma, Vulnerability and Palestinian Children under Occupation

Twenty years after Pupavac provocatively argued that “[T]rauma is displacing hunger in the West’s conceptualisation of the impact of wars and disasters in the South”, psychosocial programming has diversified in its forms but its central role in humanitarian work with children endures ([29,37] p. 358). Over recent decades, the occupied Palestinian territory of East Jerusalem, the West Bank and Gaza, has witnessed an abundance of such programming delivered by international NGOs, UN agencies and local organisations [38]. Much of this work has been directed at children, or at adults in their role as children’s caregivers. For the institutions involved, psychosocial intervention constitutes a logical response to the assessment of children’s most pressing needs. The incidence of trauma (PTSD), measured through a variety of means, has been commonly invoked as part of such assessment [39,40].

Situations regularly encountered by Palestinian children that might have severe adverse effects upon their mental health and wellbeing are numerous. They include targeting for violent attack by Israeli forces and settlers; being arrested and held in administrative detention under conditions that violate international law; denial of family reunification; obstruction in accessing basic services; and expropriation or destruction of their homes. Notwithstanding this litany of violations that Palestinian children routinely experience, their conceptualisation as traumatised victims is reductive. It ignores the roles that they continue to play in Palestinian society even amidst the violence and oppression, as workers, caregivers, young parents, activists, builders of communal identity, humanitarians, and resistance fighters [41,42,43]. In short, they are protagonists in the terms suggested by Liebl: “economic and social subjects contributing to the development of their community and country” ([44] p. 70). Indeed, in various ways the conditions of occupation and blockade may expand the engagement of the young as social, economic, and political actors even while they deal with the harms inflicted upon them.

The reductive conceptualisation of Palestinian children as traumatised victims is maintained by various factors, including the vogue within child-focused humanitarianism for psychosocial programming. Naturalised as a response to violations, such programming is given primacy over other potential actions that UN agencies and international NGOs might take, most particularly efforts to pressure Israel to abide by its obligations under international humanitarian and human rights law.

The privileging of psychosocial response and the enduring invocation of children’s vulnerability that serves to justify a primary focus on mental health, must be situated in relation to the pursuit of political and economic interests by powerful players both within and far distant from the setting itself. They include western donor governments, international and UN humanitarian agencies, multinational corporations, as well as the Government of Israel. As I shall explain, the conceptualisation of Palestinian children as vulnerable, traumatised victims serves the interests of each of these.

The Israeli authorities routinely portray adult Palestinians as ‘terrorists’. By contrast, Palestinian children are victims vulnerable to the brainwashing and exploitation of the political leadership who seek to turn them into ‘terrorists’. This pathologising view is found even in some of the academic literature and the remedy, implied or made explicit, is that intervention is needed to rescue Palestinian children from the nihilism and violence of their own society [21,45]. Such ‘rescue’ may take different forms, including efforts to alter Palestinian school textbooks on the highly questionable grounds that they are responsible for inculcating anti-Israeli sentiment amongst school students [46]. The impact upon the development of political consciousness of children’s routine exposure to diverse forms of violence by a foreign power is commonly ignored.

Western governments have fully aligned themselves with successive Israeli administrations: developing collaboration in a number of sectors, most importantly security and defence, where joint efforts to develop weaponry and surveillance equipment have proven extremely lucrative [47,48,49]. The need to protect these arrangements from scrutiny and criticism is reflected in the constraints that western donor governments are liable to impose upon humanitarian agencies that they fund. Such constraints have included making support conditional upon the commitment not to endorse non-violent forms of action, most notably the Boycott, Divestment and Sanctions campaign mounted by Palestinian civil society to challenge Israeli occupation [50,51].

The influence of donors operates at a systemic level. After decades of work in this part of the world, UN agencies and INGOs have internalised the expectations of their funders. Staff at HQ level are likely to police the speech and actions of employees working on the ground. In a study conducted by Hart and Lo Forte, a Jerusalem-based employee of UNICEF is quoted as follows: “We had more pressure on what we could say about violations of children’s rights from New York [HQ] than even from the Israeli government.” ([52] p. 31)

Child-focused humanitarian INGOs and UN agencies walk a tightrope. They are formally tasked with the responsibility to ensure the protection of Palestinian children. Yet, at the same time, their funding and, potentially their presence on the ground, depend in large measure on avoiding disturbance of political and economic relations between the government of Israel and western governments. Moreover, it is not only support for humanitarian work in the oPt that is at stake. Conflict with funders could put at risk an organisation’s finances around the globe.

In a marketplace that has grown more crowded with the arrival of new players, and as governmental donors cut their funding, the financial stakes for the established players-INGOs and UN agencies–have grown greater. In addition, child-focused humanitarian organisations operating in the oPt have had to consider the sensitivities of corporate donors as private sector support grows in importance. Over several years, UNICEF NYHQ received funds from Caterpillar. (Announcement of three-year funding from Caterpillar Foundation to UNICEF Available online: https://www.unicefusa.org/supporters/organizations/companies/partners/caterpillar-foundation (accessed on 23 February 2023))—a company that manufactures bulldozers used by the Israeli army to demolish Palestinian homes. Meanwhile Save the Children US (SCUS) received financial support from foundations linked directly to Hewlett Packard which developed the computer technology used at Israeli checkpoints. (Announcement of funding from the William and Flora Hewlett Foundation to Save the Children USA. Available online: https://hewlett.org/grants/save-the-children-for-reproductive-health-activities/ (accessed on 23 February 2023)). Even though neither company seems to have supported humanitarian work in the oPt, it is reasonable to raise concerns about a conflict of interests for organisations mandated to undertake child protection work that are in receipt of funds from companies that have contributed to the harm done to Palestinian children.

The approach conventionally taken by child-focused humanitarians in the oPt relates closely to the political and economic agendas just outlined. Conceptualisation of children as vulnerable and traumatised victims serves to justify interventions that treat the young as objects of care rather than as subjects of rights with the capacity to speak and act for the realisation of those rights. Thus, while the enduring abundance of psychosocial programming in the oPt is, in part, motivated by global trends within the humanitarian field, it has also been a means to legitimise interventions predicated on an ethos of therapeutic governance. Rather than the young being seen as aware, capable social and political actors with grievances and aspirations worthy of serious attention, they are cast as fragile and damaged. There is no inherent reason that the young here, as elsewhere, cannot be both at the same time. However, in the oPt the latter is conventionally highlighted and the former downplayed, if not ignored.

As a sector child-focused humanitarian organisations share a commitment to take the views of children meaningfully into account. In the oPt, however, there is considerable risk for humanitarian organisations engaging the young in activities that allow for articulation of experiences of growing up under occupation and discussion of aspirations for liberation. Over the years, young Palestinians have mobilised to advocate for themselves, often making explicit criticism of organisations mandated to work for their protection and wellbeing. Various petitions have been produced, and artistic and journalistic initiatives launched through which Palestinian children have sought to engage an international audience. For example, in 2002 a group of children in Gaza involved with a ‘Young Parliament’, under the auspices of a local NGO, created a petition that called upon the international community to fulfil its obligations for their protection. This was signed by 20,000 Palestinian children and delivered to Mary Robinson, then UN High Commissioner for Human Rights, during her visit to Gaza. [53]. (Other initiatives include a photo essay by a group of 14–25-year-old volunteers with a Palestinian youth organisation, published after some frank debate with journal editors evidently nervous about the content. PYALARA 2007) [54].

Initiatives driven by young Palestinians stand in stark contrast to the activities pursued by the leading humanitarian organisations for whom ‘participation’ is managed carefully, with strong emphasis placed upon remedial efforts to address trauma and harm done. It was striking, for example, that in the years immediately prior to its departure from the occupied Palestinian territory, Save the Children US, conflated ‘child protection’ and ‘psychosocial’ programming, as if Palestinian children could be protected from the various violations inflicted by the Israeli state and settlers through attention to their mental health. (Save the Children US no longer works in the occupied Palestinian territory. However, back in 2010 when it was active on the ground their website explained their approach to child protection (a priority area for that organisation) entirely in terms of psychosocial programming) [51].

Open-ended engagement with the young may bring the demand for justice centre-stage. It is far safer for humanitarian organisations such as SCUS to focus on children’s vulnerability and their need for healing. Even the UK branch of Save the Children, that has shown more readiness to speak openly about Israeli state violence, generally shies away from engagement with Palestinian children as protagonists in pursuit of justice. There is a familiar format—witnessed in a recent report on home demolitions—in which children are involved in articulating their experience as victims of a particular form of violation by the Israeli authorities and extremist settlers [55]. However, initiatives in which the young are encouraged to share their experiences, articulate their expectations of humanitarian organisations, and talk about their aspirations in relation to the struggles of their communities and wider society are not the standard fare of INGOs and UN agencies.

## 9. Conclusions

I began my discussion in the industrialising nations of the 18th Century and ended in the present-day setting of the occupied Palestinian territory. Across this sweep of history and geography, a conceptualisation of the child as vulnerable victim has emerged and come to dominate the discourse and practice of western-originating humanitarian organisations. Without denying the vulnerability that children may experience, I have sought to question its automatic invocation as the primary defining characteristic. As I have acknowledged, such invocation may yield certain benefits for children in certain settings: for example, by ensuring that they receive more compassionate treatment.

Nonetheless, it is my contention that the system-wide tendency to identify all young people living amidst humanitarian crisis as inevitably vulnerable by virtue of the context is an obstacle to effective protection work. Frequently asserted through allusion to the mental health situation of the young (as traumatised victims), ascription of vulnerability serves as a boundary marker for engagement. The roles and responsibilities normally undertaken by children in much of the majority world are likely to increase in the context of displacement and political violence: families are dispersed, economic survival becomes harder, conventional caregivers are incapacitated, and the young become active in the struggle for justice or liberation. Engaging children about the reality of their lives, rather than around issues deemed appropriate to vulnerable victims, is essential if protection efforts are to be relevant and supportive.

While there has been increasing attention to children’s agency in recent years, this has not provided the needed corrective to the crude understanding of vulnerability that has dominated thinking and praxis amongst humanitarians. Instead, it has been subordinated to a simplistic dichotomy in which children are either vulnerable victims or resilient social actors. Rather than reducing individuals to one amongst a series of possible archetypes—the trafficking victim, the child soldier, the child bride, the resilient survivor, and so on—we need an approach that considers children’s lives as a whole, exploring the roles that they undertake, the risks that they negotiate, the harms that they experience, and the strategies that they employ to enhance their own protection and that of others.

There is also a need to challenge assumptions of vulnerability as inherent to the young by virtue of their chronological age: considering instead how children in a given context are rendered vulnerable. Central to such analysis is disaggregation of the category of ‘child’, employing an intersectional lens. This entails thinking not only about differences between, for example, older and younger children but also how those of the same age might be rendered vulnerable differently due to the mediating impact of gender, ethnicity, class, and so on. Moreover, analysis needs to relate the production of vulnerability to the environment, attending to the effects, inter alia, of patriarchy, colonialism, and racism.

In this paper, I have offered two specific examples that call into question the assumption that attribution of vulnerability is a politically innocent act. SCF’s involvement with the British colonial response to the Mau Mau rebellion in the early 1950s and the ongoing humanitarian response to the situation of Palestinian children living under Israeli occupation both exemplify what we might call the politics of pathologisation. They indicate how the invocation of vulnerability provides a rationale for not supporting the young by seeking to prevent violations and suffering. Focus is placed on healing the harm caused by violent and oppressive forces while ignoring the cause of such harm. Meanwhile, the interests of corporate actors, political elites as well as international non-governmental and UN agencies are protected.

Given the political and economic agendas at stake, there is scant incentive for humanitarians to reflect upon and revise an understanding of vulnerability that has hitherto helped to maintain a fragile status quo. Dialogue on the ways that the vulnerability of children is invoked and addressed is unlikely to open up without pressure from beyond the professionalised field of child-focused humanitarianism. The task is, indeed, a societal one. It entails moving beyond the stasis in dominant conceptualisation of the child in humanitarian settings as an inevitably vulnerable victim. Such stasis is, in itself, bizarre when compared to the contestation and change concerning the perceived capacities and roles of women. The conceptualisation of children not residing in humanitarian settings has been the focus of lively debate in the global North for at least the last forty years. Yet, the perception of refugee children and those living in settings of political violence as primarily vulnerable has, if anything, become more entrenched. Far from countering this trend, the focus upon children’s agency and participation has contributed to it. Reflexive consideration of the lived experience of young people as vulnerable victims, as social actors and as neither and both is essential and long overdue.

## Data Availability

The data presented in this study are available on request from the author. The data are not publicly available due to concerns for the privacy and security of research participants.
